# Psychometric evaluation of the desire to avoid pregnancy scale in India^☆^

**DOI:** 10.1016/j.contraception.2025.110940

**Published:** 2025-05-08

**Authors:** Sarah Averbach, Nicole E. Johns, Shweta Tomar, Marielle E. Meurice, Namratha Rao, Mohan Ghule, Anita Raj

**Affiliations:** a Center on Gender Equity and Health, Department of Medicine, Division of Infectious Disease and Global Public Health, University of California, San Diego, La Jolla, CA, USA; b University of California, San Diego, Department of Obstetrics, Gynecology, and Reproductive Sciences, Division of Complex Family Planning, La Jolla, CA, USA; c Center on Gender Equity and Health, University of California, San Diego, India office, Maharashtra, India; d Newcomb Institute, Tulane University, New Orleans, LA, USA; e Tulane University School of Public Health and Tropical Medicine, New Orleans, LA, USA

**Keywords:** Contraception, Family planning, Pregnancy intendedness, Psychometric evaluation

## Abstract

**Objective:**

This study aimed to evaluate the psychometric performance of the Desire to Avoid Pregnancy (DAP) scale in India.

**Study design:**

We utilized survey data from married women enrolled in a family planning intervention in Maharashtra, India, who provided responses to the 14-item DAP scale at 18-month intervention study follow-up. We assessed scale internal consistency using Cronbach α and used exploratory factor analysis to evaluate scale unidimensionality and item response theory (IRT) to assess item performance. We used regression models to assess whether DAP predicts current reported contraceptive use, as well as future contraceptive use and pregnancy, to evaluate construct validity.

**Results:**

A total of 1088 participants responded to 18-month intervention study follow-up survey; 99% of eligible participants (887/888) provided the full-scale response. One item, “makes me smile,” performed in the reverse direction as anticipated (negative item-test correlation) and was excluded for use in this analysis. The mean 13-item DAP score (DAP-13) was 2.14 of 4 (SD 0.95, range 0–4); internal consistency was high (Cronbach α = 0.92). Most items fit the partial credit model on IRT. Exploratory factor analyses supported either a one- or two-factor model; the unidimensional model was considered acceptable for use as the single factor explained 71% of all variance, and all items had stable absolute factor loadings ≥0.38. DAP-13 score only differed by parity; nulliparous women had the lowest scores (0.56), followed by women with one (1.94), two (2.60), or three or more births (2.56; p < 0.001). A one-point increase in DAP-13 was associated with greater odds of current contraception use reported at time of DAP assessment (adjusted odds ratio [aOR] 1.79, 95% CI 1.43–2.26), subsequent contraception use reported 18 months after DAP assessment (aOR 1.88, 95% CI 1.44–2.44), and half the odds of subsequent pregnancy in the 18-month period after DAP assessment (aOR 0.57, 95% CI 0.46–0.71).

**Conclusions:**

The DAP scale demonstrated good reliability and unidimensionality in this population. Higher DAP scores were associated with higher odds of contraception use and lower odds of pregnancy, supporting construct validity. Future research on the DAP scale in Maharashtra should explore alternative translations of the “makes me smile” item that better capture local expressions of joy about potential childbearing.

**Implications:**

A modified version of the DAP scale is acceptable for use in an Indian population and can be utilized in future research and program evaluation that focus on contraception and pregnancy prevention in this context. One item was excluded and requires additional formative research to better capture the intended emotional valence in this cultural context.

**Clinical trial registration number:**

NCT03514914

## 1. Introduction

Understanding and measuring pregnancy intention are important to support preference-sensitive contraceptive counseling and care and to assess whether family planning interventions meet the needs of users.

Pregnancy intention is typically assessed retrospectively, with people asked to recall their desire to become pregnant at the time of conception. Globally, Demographic and Health Surveys, widely used standardized nationally representative surveys implemented in many low- and middle-income country (LMIC) settings, use the item “When you got pregnant, did you want to get pregnant at that time?”, with those responding “no” further asked “Did you want to have a baby later on, or did you not want any [more] children?” [1]. Such retrospective reporting is prone to bias, with respondents consistently overreporting that a pregnancy was intended, particularly when the pregnancy resulted in a birth rather than a termination [2–4]. Future fertility intention is often assessed by asking whether respondents would like to have a(nother) child, and if yes, and how long they would like to wait [1]. However, many people feel uncertain or have ambivalence about pregnancy [5,6], and simple binary measures fail to capture the complexity of pregnancy intentions. Assessing pregnancy intendedness using a more nuanced, prospective, continuous measure can enable clinicians, policymakers, and researchers to better understand family planning needs and tailor interventions to be more person centered.

The 14-item Desire to Avoid Pregnancy (DAP) scale was developed to assess preferences about a future pregnancy by asking about a hypothetical pregnancy within the subsequent 3 months and birth within the subsequent year [7]. It includes three conceptual domains (cognitive preferences, affective feelings, and practical consequences). The DAP scale was developed in the United States and has been studied and validated in the United States, the United Kingdom, and Turkey [8–11]. The scale has been associated with current and future contraceptive use behaviors and with the likelihood of subsequent pregnancy in these settings [9,12,13]. While there is ongoing research validating and utilizing DAP in other countries [14], there is no published data that we are aware of from India or any other LMIC settings [15].

We aimed to assess the validity of the DAP scale in India, the most populous country in the world and a nation with a large number of people with an unmet need for contraception [16]. Efforts aimed at improving the quality of family planning care in India have the potential to better meet the reproductive needs of many people while ensuring their reproductive autonomy. It is important to assess the validity of using measures such as DAP in India and other LMIC settings to allow for efficient and effective assessment of the need for family planning programs and to track the effects of quality improvement activities. We implemented the DAP scale in the context of an intervention trial to measure pregnancy intention in a way that better captures the ambiguity of how people conceptualize pregnancy and the diverse feelings people have about a potential pregnancy.

## 2. Materials and methods

This study utilizes two waves of follow-up data from married women enrolled in the Counseling Husbands and wives to Achieve Reproductive health and Marital equity (CHARM2) study. This two-arm, clustered, randomized controlled trial (clinical trial number NCT03514914) evaluated a family planning and gender equity counseling intervention in Maharashtra, India. Study methods are provided in detail elsewhere [17,18]. Briefly, data collection occurred at baseline and 9, 18, and 36 months after. We analyzed data from surveys conducted at the 18- and 36-month follow-ups, conducted from June to December 2020 and March to August 2022, respectively. Prior survey waves were not utilized, as DAP was only included on the 18- and 36-month follow-up. DAP was not measured at baseline or 9-month follow-up.

Inclusion criteria for CHARM2 included married couples with women aged 18 to 29 years, who had not undergone permanent female contraception, and were living together for at least 6 months. We recruited via random selection from household rosters and approached and interviewed participants in their homes. Any female participant who was not pregnant at 18-month follow-up was eligible to complete the DAP scale.

### 2.1. DAP scale and adaptation

The original DAP scale includes 14 items, each with a 5-point Likert scale response from 0 “strongly agree” to 4 “strongly disagree”. Five items ask about feelings toward pregnancy in the next 3 months, and nine ask about feelings toward having a baby in the next year. The score is summarized as an average response to the 14 items, ranging from 0 to 4, with higher scores representing stronger desire to avoid pregnancy. Half of items are reverse coded. DAP is intended to be used as a continuous measure (Appendix Table A).

We translated DAP into Marathi, the local language of the study area. Translation was performed by one local study staff member fluent in both English and Marathi. Translation and back-translation were reviewed and modified by additional local staff members who speak both languages; items were then pilot tested with 10 women in the study area to assess comprehension and acceptability. Items were well understood and acceptable; no substantive changes to prompts, scale items, or response options were made.

### 2.2. Outcomes for construct validity testing

To text construct validity by hypothesis testing, we examined the association between DAP scores and outcomes that we hypothesized are likely to related to desire to avoid pregnancy including contraceptive method use and subsequent pregnancy.

Contraceptive use was defined at each time point based on self-reported method use within the prior 3 months. “Modern” methods reported by women in the study included condoms, pills, intrauterine devices, injectables, and emergency contraception [19]. “Any” contraceptive method includes modern methods as well as fertility awareness, withdrawal, lactational amenorrhea, and abstinence [19].

Subsequent pregnancy was defined as any self-reported pregnancy between 18- and 36-month surveys. Women were categorized as having had a subsequent pregnancy if they reported current pregnancy at 36-month follow-up, a pregnancy of any outcome occurring in the year prior to 36-month follow-up, or if they reported a higher number of total births at 36 months than at 18 months. As these surveys were conducted approximately 18 months apart and the 36-month survey assessed only pregnancies occurring in the prior year, a small number of pregnancies that began and ended within the 6 months after the 18-month survey (e.g., abortions, miscarriages) may not be captured.

### 2.3. Sample size and statistical analyses

It is estimated that 2 to 20 subjects per item are required for psychometric validation [20]; therefore, we estimated that we would require no more than 280 participants for this analysis. The number of respondents to the full DAP scale at 18-month survey (*n* = 887) was thus expected to be more than sufficient to complete this analysis.

We assessed internal scale reliability via Cronbach α test (if an item is poorly correlated with the total score, it suggests that item decreases the reliability of the scale). We used exploratory factor analysis to evaluate scale unidimensionality or whether all scale items capture one construct rather than multiple separate constructs. We used classical test theory, including exploratory factor analysis with a polychoric matrix to conduct the primary analysis, because it is a broadly used and understood method and allows for comparison to prior psychometric analyses of DAP [8]. We used Kaiser’s criteria (eigenvalue > 1) to identify potentially relevant factors and examined Scree plots and percent of variance explained to consider the number of factors to be retained and absolute factor loadings of approximately 0.4 or greater to indicate stable loading on to a relevant factor [21,22]. We were particularly interested in examining whether utilizing the scale as a unidimensional measure would be appropriate to maintain comparability with scale development, validation, and previous utilization. Therefore, we considered that if all items loaded at 0.4 or greater onto a single factor with an eigenvalue greater than one and greater than 50% of variance explained, the scale would be considered appropriate to be considered unidimensional. Additionally, as the DAP was originally developed using item response theory (IRT), we conducted an IRT analysis using the same methodology for comparability [7]. We fitted all items to a partial credit model and tested for item fit using weighted mean square index of 0.67 to 1.33 as a good fit [7]. We plotted the item characteristic curves to assess monotonicity. We plotted and examined the Wright map—a map of women’s overall scores on the scale next to item threshold difficulty levels—to test how items and item thresholds fell relative to overall score.

We assessed DAP scores for the study population as a whole and by key sociodemographic measures. We assessed the distribution of the DAP scale to ensure assumptions of normality were met. We used *t* tests to assess for bivariate differences in DAP score for binary measures and analysis of variance for categorical measures and Spearman correlation for continuous measures. We used mixed effects multivariate logistic regression analyses to test construct validity and examine the association between DAP score and current contraceptive use (use at 18-month study follow-up, the time of DAP assessment) and future contraceptive use (use at 36-month study follow-up). We included both unadjusted models and models adjusted for sociodemographic factors, which we selected *a priori* due to associations with family planning outcome (parity, age) or associations with study treatment condition and/or loss to follow-up. We assessed for and found no evidence of multicollinearity in adjusted models; all covariates were retained. All models included random intercept specifications for subcenter, which was the geographic unit of randomization in the CHARM2 study and reflects the catchment area of a public health facility.

Finally, we assessed associations between DAP scale score at 18-month follow-up and subsequent pregnancy utilizing mixed effects logistic regression for unadjusted and adjusted models, including random intercept specifications for subcenter.

### 2.4. Sociodemographic characteristics

We included several sociodemographic characteristics in the models selected a priori based on established associations with contraceptive use outcomes or which differed significantly by CHARM2 treatment status and/or loss to follow-up. We analyzed age in years as a continuous measure and parity grouped as 0, 1, 2, or 3 or more births. We defined child marriage as occurring before the age of 18 years. We categorized education as primary or no education, secondary, or more than secondary education. We coded religion as a binary, Hindu vs other religions (which include Muslim, Buddhist, Jain, Christian, or other). We included a binary indicator for “scheduled tribe, scheduled caste, or other backwards class” designation. These are official government designations of historic disadvantage, which are linked to governmental policies and interventions. We also included an indicator of household ownership of a “Below Poverty Line card”, a proxy for low income. We included a binary indicator of whether the woman’s mother-in-law resides in the same household. Age was calculated based on the date of birth provided at baseline survey and date of 18-month survey. Parity was directly assessed at 18-month survey. All other sociodemographic variables were based on responses to the baseline survey.

Analyses were conducted using Stata 15.1 (StataCorp, College Station Texas) and R version 4.3.3 (R Core Team). Statistical significance was set at *p* < 0.05; We reported 95% CIs for odds ratios (ORs) and adjusted odds ratios (aORs).

### 2.5. Ethical review

We obtained Ethical approval from the Institutional Review Boards of the National Institute for Research in Reproductive and Child Health Ethics Committee, Population Council, and Sigma in India, and the University of California, San Diego, in the United States. Written informed consent was obtained from all participants for each survey.

## 3. Results

### 3.1. Sample characteristics

At 18-month intervention study follow-up, 1088 women responded, 91% retention from baseline (*n* = 1201); 888 were nonpregnant and had not undergone permanent contraception and thus eligible to respond to the DAP scale. Of these, 887 (99%) provided the full DAP scale response and comprise the primary sample; only one individual declined one DAP scale item and was thus removed from DAP scale analyses. Of the 887 women with valid DAP scale responses, 828 responded to the 36-month survey and constitute the sample for analyses of the association between DAP and subsequent contraceptive use and pregnancy.

Women were 26 (range 19–31) years old, on average (Table 1). One in six (16%) were married before the age of 18 years. Most (59%) had at least a higher secondary education, and (92%) were Hindu. About one-third (30%) belonged to a “scheduled tribe, scheduled caste, or other backwards class.” One-quarter (23%) reported that their household held a Below Poverty Line card. Most (80%) lived in the same household as their mother-in-law and had given birth one time (53%).

### 3.2. DAP scale score reliability, unidimensionality, and sociodemographic associations

The mean DAP scale score at 18-month survey was 2.21 out of 4 (SD 0.86, range 0–4); the mean score of each individual item ranged from 0.88 to 3.07 (Appendix A Table). Absolute item-test correlations were moderate to strong for all examined items, ranging from 0.40 to 0.84, except one item, “Thinking about having a baby within the next year makes me smile,” which performed in the reverse direction as anticipated (negative item-test correlation) due to an interpretational/translational issue discovered after survey administration (see Discussion) and was dropped for future analysis. The mean 13-item DAP scale (DAP-13) score was 2.14 out of 4 (SD 0.95, range 0–4; Appendix C). Item-test correlations were moderate to strong for all examined items, ranging from 0.45 to 0.82 (Appendix B Table).

The full DAP scale reliability was high overall (Cronbach α = 0.90). Cronbach α remained similar when any single item was removed (α if removed ranging from 0.89 to 0.92). Removal of the single item, DAP-13, “Thinking about having a baby within the next year makes me smile” led to slightly higher α = 0.92 (and α if removed ranging from 0.91 to 0.92).

Exploratory factor analyses supported the DAP-13 scale’s unidimensionality. Though two components had eigenvalue greater than 1 (single-item eigenvalue 6.57, second-item eigenvalue 2.45), a high percentage of variance was explained by the single factor (71%) and the absolute value of factor loadings on the single factor was high, ranging from 0.38 to 0.85. Additionally, the items that loaded on to the second factor did not group together in any clear theoretical way (e.g. negatively vs positively worded items or initially developed domains of the DAP). This ultimately suggests that the DAP-13 items acceptably capture a single underlying construct and that all 13 items are reasonably related to that underlying construct.

The IRT results (Appendix D Table) show that DAP-13 has a separation reliability of 0.93. Based on the weighted mean square of 0.67–1.33, most items fit the partial credit model, but two items did not. Consistent with the original DAP scale development, the item “Becoming pregnant in the next 3 months would bring me closer to my husband” had the poorest fit followed by “It would be the end of the world for me to have a baby in the next year.”

Monotonicity was confirmed using item characteristic curves (not shown). The Wright map (not shown) demonstrated that the items covered a broad range of the latent construct (−4 to +4 logits). The item threshold locations aligned well with the participant DAP score levels with clustering of thresholds in the middle range (−1 to +1 logits) and sparse coverage at the extremes. Some clustering of response categories (category 2 and category 3) suggests that few participants in this context answered items reporting that they “neither agree nor disagree” (Appendix E Table).

DAP-13 score did not differ by most sociodemographic factors, differing significantly only by parity, with nulliparous women have a lower DAP-13 score (0.56), followed by women with one (1.94), two (2.60), or three or more births (2.56; *p* < 0.001; Table 2).

### 3.3. Association between DAP-13 and current and future contraception use

Mean DAP-13 scores were significantly different between users and nonusers of any contraception (2.28 vs 1.54, *p* < 0.001; OR 2.29, 95% CI 1.90–2.77), and modern contraception (2.32 vs 1.96, *p* < 0.001; OR 1.49, 95% CI 1.29–1.73) at 18 months and any contraception (2.31 vs 1.48, *p* < 0.001; OR 2.56, 95% CI 2.05–3.19) and modern contraception (2.34 vs 1.96, *p* < 0.001; OR 1.55, 95% CI 1.33–1.82) at 36 months. A one-point increase in 18-month DAP-13 score was associated with greater odds of current contraceptive use (any [aOR 1.79, 95% CI, 1.43–2.26] and modern [aOR 1.42, 95% CI 1.20–1.69] use, respectively; Table 3). Similarly, DAP-13 score at 18 months was associated with future contraceptive use at 36 months (both any [aOR 1.88, 95% CI 1.44–2.44] and modern [aOR 1.38, 95% CI 1.14–1.67] contraception, respectively).

### 3.4. DAP and pregnancy

**Fig. 1.**
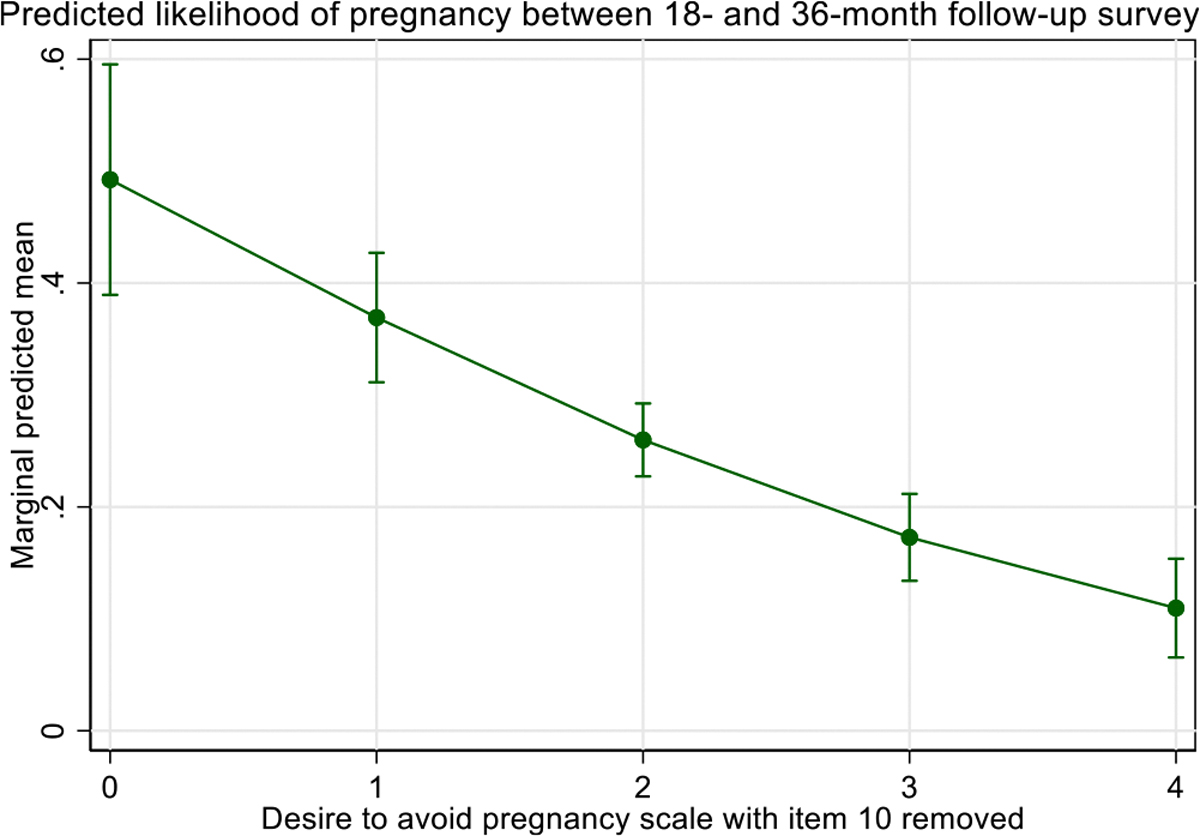
Likelihood of pregnancy and previous DAP score in Maharashtra, India, 2020–2022 (*N* = 887).

DAP-13 score at 18 months differed between those who did and did not have a subsequent pregnancy at 36 months (1.71 vs 2.30, *p* < 0.001; OR 0.51, 95% CI 0.43–0.61). A one-point increase in 18-month DAP-13 score was associated with half the odds of having a subsequent pregnancy, adjusting for sociodemographic characteristics including parity (aOR 0.57, 95% CI 0.46–0.71; Table 3, Figure 1).

## 4. Discussion

We found that the DAP scale may be appropriate for use in a rural Indian population. After removal of a poorly performing item, we found the DAP-13 scale has good reliability and unidimensionality and evidence of construct validity with related outcomes (higher odds of family planning use and lower odds of subsequent pregnancy) in this population. These data suggest that this measure is suitable for use for family planning research and clinical contexts in India.

One item, “Thinking about having a baby within the next year makes me smile,” performed in the reverse direction as anticipated (negative item-test correlation). Through further discussion with the field team, we learned that the word smile may not capture the intended emotional valence in this cultural context. In fact, the word can mean both “smile” and “laugh” in Marathi. The phrasing may have been experienced as disconnected from how people naturally express happiness about having children and instead interpreted as an uncomfortable negative feeling about childbearing. Therefore, we conducted our analyses without that item. We recommend future use of the scale in Marathi language include a more careful translation or explanation of “makes me smile” that is better aligned with the intended meaning or that the item be adapted to develop and test a more context-appropriate positive affective item that is more culturally resonant through formative research on local expressions of joy about potential childbearing.

Similar to what was seen in other validated contexts [8,11], we found a high Cronbach alpha (0.92) suggesting high reliability in this setting. We also found a mean DAP score (2.21) that is similar to that of people surveyed in reproductive and primary care settings in the United States and the United Kingdom (2.2–2.5) [8,12,23,24].

The observed association between higher DAP-13 scores and greater use of contraception is aligned with the theoretical pathway that people who have a stronger intention to avoid pregnancy are more likely to use contraception. Most studies have found a similar association [8,12,13,24]. We were also able to demonstrate an association between DAP-13 score and subsequent pregnancy, which is consistent with theoretical pathway and prior data that people who have a stronger intention to avoid pregnancy are less likely to become pregnant [8,9]; however, fewer studies have had the power and longitudinal follow-up to assess pregnancy outcomes.

Unlike the studies in the United States and the United Kingdom, we did not find a cluster of participants falling around the lowest and highest DAP score levels (0 and 4) [7,8]. Similar to findings in the United Kingdom, we found that increasing number of children is associated with DAP [8]. In the United States, parous participants overall had slightly lower DAP scores than nulliparous participants [12]. In our study, poverty was not associated with DAP, whereas it was in the United States (among participants in the lower quartile of scores) [12]. We did not find that age was associated with DAP in India, though it was in the United Kingdom [8].

The strengths of our study include high follow-up rates, rigorous sampling, large sample size, and longitudinal follow-up allowing for assessment of the association between DAP-13 and pregnancy.

The limitations include that no qualitative work or formal cognitive interviews were done to evaluate the measures in the context to support construct validity. This work was, however, done in the context of our focus groups and semistructured interviews with participants in this and other studies in the region during the same time period to understand norms and attitudes toward family planning and pregnancy. These data suggest that traditionally, women in the community are expected to demonstrate fertility early in marriage and not use contraception until the number and desired sex (sex assigned at birth) of children is achieved [25]. However, social norms are changing, and married women in India feel they have greater control over their fertility and reproductive decision-making than in the past [26]. Findings from this study also suggest that women are the primary decision makers in whether to use contraception [27]. There is a cultural focus on achieving healthy birth spacing and avoiding pregnancy soon after birth by using contraception [25]. Still, one item performed in the reverse direction as anticipated and further cognitive interviewing and subsequent cohort testing could be done to understand that item in the context of rural India or if an adapted item would be more appropriate in this setting.

Our generalizability is limited to married women in Maharashtra, India, and, to some extent, rural India. We tested the association between the DAP scale at 18 months and contraceptive use at 36 months because there was no data collection between those two surveys. It is possible that DAP scores could change meaningfully over that time. Nevertheless, we found an association between DAP score and subsequent contraceptive use and pregnancy. Finally, we did not assess test-retest reliability and perform confirmatory factor analysis on a second sample.

In summary, we found that the DAP scale may be a valid and reliable measure of desire to avoid pregnancy in India, an LMIC setting. In addition, this is one of the few studies to show that the DAP score is predictive of pregnancy. Future research should consider validation of a short form to enhance use in clinical settings preceded by cognitive interviewing.

## Supplementary Material

1

## Figures and Tables

**Table 1:** Sample characteristics of women providing DAP scale response at CHARM2 18-month follow-up survey in Maharashtra, India, 2020–2022 (*N* = 887)

Characteristic	*n* (%)

Age (continuous), mean years (SD)	26 (3)
Married before age 18 y	
Yes	139 (15.7)
Education	
Primary or no education (0–8)	105 (11.8)
Secondary (9–10)	255 (28.7)
Higher secondary or more (11+)	527 (59.4)
Religion	
Hindu	820 (92.4)
Muslim/Buddhist/Jain/Christian/Other	67 (7.6)
SCST designation	
None/other	618 (69.7)
SC/ST/OBC	269 (30.3)
Household has BPL card	
Yes	205 (23.1)
Mother-in-law lives in same household	
Yes	726 (81.8)
Parity	
0	46 (5.2)
1	471 (53.1)
2	317 (35.7)
3+	53 (6.0)

BPL, below poverty line; DAP, desire to avoid pregnancy; OBC, other backwards class; SC, scheduled cast; ST, scheduled tribe.

**Table 2:** Average DAP-13 scale score by sociodemographic factors in Maharashtra, India, 2020–2022 (*N* = 887)

Characteristic	Mean DAP-13 score (SD)	*p*-value

Overall	2.14 (0.95)	
Married before age 18 y		0.26
No	2.16 (0.96)	
Yes	2.06 (0.94)	
Education		0.32
Primary or no education (0–8)	2.04 (0.92)	
Secondary (9–10)	2.10 (0.98)	
Higher secondary or more (11+)	2.18 (0.95)	
Religion		0.62
Hindu	2.14 (0.96)	
Muslim/Buddhist/Jain/Christian/Other	2.08 (0.91)	
SCST designation		0.69
None/other	2.13 (0.95)	
SC/ST/OBC	2.16 (0.96)	
Household has BPL card		0.65
No	2.15 (0.94)	
Yes	2.11 (0.99)	
Mother-in-law lives in same household		0.99
No	2.14 (0.98)	
Yes	2.14 (0.95)	
Parity		< 0.001
0	0.56 (0.51)	
1	1.94 (0.88)	
2	2.60 (0.77)	
3+	2.56 (0.82)	

BPL, below poverty line; DAP, desire to avoid pregnancy; OBC, other backwards class; SC, scheduled cast; ST, scheduled tribe.

**Table 3:** Association between 18-month DAP-13 score and current and future contraceptive use and pregnancy in Maharashtra, India, 2020–2022 *(N =* 887)

Contraceptive use and pregnancy	Mean DAP-13 score		Unadjusted			Adjusted^a^		

			OR	95% CI	*p*-value	aOR	95% CI	*p*-value
Current contraceptive use	Use	Nonuse						
Any contraception	2.28	1.54	2.29	1.90–2.77	< 0.001	1.79	1.43–2.26	0.001
Modern contraception	2.32	1.96	1.49	1.29–1.73	< 0.001	1.42	1.20–1.69	< 0.001
Future contraceptive use	Use	Nonuse						
Any contraception	2.31	1.48	2.56	2.05–3.19	< 0.001	1.88	1.44–2.44	< 0.001
Modern contraception	2.34	1.96	1.55	1.33–1.82	< 0.001	1.38	1.14–1.67	0.001
Future pregnancy	Yes	No						
	1.71	2.30	0.51	0.43–0.61	< 0.001	0.57	0.46–0.71	< 0.001

aOR, adjusted odds ratio; DAP, desire to avoid pregnancy; FP, family planning; OR, odds ratio.

a Adjusted models controlled for age, age at marriage, education, religion, scheduled tribe/scheduled caste designation, household below poverty line card ownership, mother- in-law living in same household, and parity. All models account for subcenter geographic clustering via random intercept specifications.

## Appendix A. Supporting information

Supplementary data associated with this article can be found in the online version at doi:10.1016/j.contraception.2025.110940.

## References

[1] Demographic and Health Surveys. Demographic and Health Surveys: Women’s Questionnaire. 2019. Available:https://dhsprogram.com/publications/publication-DHSQ8-DHS-Questionnairesand-Manuals.cfm. Accessed August 15, 2024.

[2] Rocca CH, Wilson MR, Jeon M, Foster DG. Stability of retrospective pregnancy intention reporting among women with unwanted pregnancies in the United States. Maternal Child Health J 2019;23:1547–55. 10.1007/s10995-019-02782-9 PMC6786959 31236825

[3] Hall JA, Stephenson J, Barrett G. On the stability of reported pregnancy intentions from pregnancy to 1 year postnatally: impact of choice of measure, timing of assessment, women’s characteristics and outcome of pregnancy. Matern Child Health J 2019;23:1177–86. 10.1007/s10995-019-02748 31218607 PMC6658581

[4] Joyce T, Kaestner R, Korenman S. The stability of pregnancy intentions and pregnancy-related maternal behaviors. Matern Child Health J 2000;4:171–8. 10.1023/a:1009571313297 11097504

[5] Cutler A, McNamara B, Qasba N, Kennedy HP, Lundsberg L, Gariepy A. “I Just Don’t Know” Know”: an exploration of women’s ambivalence about a new pregnancy. Women’s Health Issues 2018;28:75–81. 10.1016/j.whi.2017.09.009 29108986 PMC6223118

[6] Schwarz EB, Lohr PA, Gold MA, Gerbert B. Prevalence and correlates of ambivalence towards pregnancy among nonpregnant women. Contraception 2007;75:305–10. 10.1016/j.contraception.2019.04.010 17362711

[7] Rocca CH, Ralph LJ, Wilson M, Gould H, Foster DG. Psychometric evaluation of an instrument to measure prospective pregnancy preferences: the desire to avoid pregnancy scale. Medical Care 2019;57:152–8. 10.1097/MLR.0000000000001048 30550399 PMC6331264

[8] Hall J, Barrett G, Rocca C. Evaluation of the desire to avoid pregnancy scale in the UK: a psychometric analysis including predictive validity. BMJ Open 2022;12:e060287. 10.1136/bmjopen-2021-060287 PMC9328097 35879004

[9] Hall JA, Barrett G, Stephenson J, Rocca CH, Edelman N. Predictive ability of the desire to avoid pregnancy scale. Reprod Health 2023;20:144. 10.1186/s12978-023-01687-9 37749640 PMC10521409

[10] Hall JA, Barrett G, Stephenson JM, Edelman NL, Rocca C. Desire to avoid pregnancy scale: clinical considerations and comparison with other questions about pregnancy preferences. BMJ Sex Reprod Health 2023;49:167–75. 10.1136/bmjsrh-2022-201750 PMC10359540 36717217

[11] Doğan Yüksekol Ö, Duman M, Timur Taşhan S. Turkish adaptation of desire to avoid pregnancy scale: a validity and reliability study. J Obstet Gynaecol Res 2022;48:431–9. 10.1111/jog.15109 34852397

[12] Samari G, Foster DG, Ralph LJ, Rocca CH. Pregnancy preferences and contraceptive use among US women. Contraception 2020;101:79–85. 10.1016/j.contraception.2019.10.007 31805265 PMC7028518

[13] Rocca CH, Smith MG, Hale NL, Khoury AJ. Ranges of pregnancy preferences and contraceptive use: results from a population-based survey in the southeast United States. Perspect Sex Reprod Health 2022;54:90–8. 10.1363/psrh.12205 36071572

[14] ANSIRH. Desire to Avoid Pregnancy (DAP) Scale [Available from: https://www.ansirh.org/research/ongoing/desire-avoid-pregnancy-dap-scale. Accessed August 15, 2024.

[15] Ren M, Shireman H, VanGompel EW, Bello JK, Carlock F, McHugh A, Preconception, interconception, and reproductive health screening tools: a systematic review. Health Serv Res 2023;58:458–88. 10.1111/1475-6773.14123 36573542 PMC10012234

[16] Sedgh G, Hussain R, Bankole A, Women with an unmet need for contraception in developing countries and their reasons for not using a method Accessed August 15, 2024 Occasional Report. Guttmacher Institute; 2007.

[17] Dixit A, Averbach S, Yore J, Kully G, Ghule M, Battala M, A gender synchronized family planning intervention for married couples in rural India: study protocol for the CHARM2 cluster randomized controlled trial evaluation. Reprod Health 2019;16:88. 10.1186/s12978-019-0744-3 31238954 PMC6593563

[18] Raj A, Ghule M, Johns NE, Battala M, Begum S, Dixit A, Evaluation of a gender synchronized family planning intervention for married couples in rural India: the CHARM2 cluster randomized control trial. EClinicalMedicine 2022;45:101334. 10.1016/j.eclinm.2022.101334 35274093 PMC8902598

[19] Hubacher D, Trussell J. A definition of modern contraceptive methods. Contraception 2015;92:420–1. 10.1016/j.contraception.2015.08.008 26276245

[20] Anthoine E, Moret L, Regnault A, Sébille V, Hardouin JB. Sample size used to validate a scale: a review of publications on newly-developed patient reported outcomes measures. Health Qual Life Outcomes 2014;12:176. 10.1186/s12955-014-0176-2 25492701 PMC4275948

[21] Kaiser HF. The application of electronic computers to factor analysis. Educ Psychol Measurement 1960;20:141–51. 10.1177/001316446002000116

[22] Izquierdo I, Olea J, Abad FJ. Exploratory factor analysis in validation studies: uses and recommendations. Psicothema 2014;26:395–400. 10.7334/psicothema2013.349 25069561

[23] Gipson JD, Bornstein M, Berger A, Rocca CH. Desire to avoid pregnancy and contraceptive use among female methadone patients in Los Angeles. Contraception 2021;103:322–7. 10.1016/j.contraception.2021.01.019 33567322

[24] Stulberg DB, Datta A, White VanGompel E, Schueler K, Rocca CH. One key question and the desire to avoid pregnancy scale: a comparison of two approaches to asking about pregnancy preferences. Contraception 2020;101:231–6. 10.1016/j.contraception.2019.12.010 31935384

[25] Dixit A, Ghule M, Rao N, Battala M, Begum S, Johns NE, Qualitative examination of the role and influence of mothers-in-law on young married couples’ family planning in rural Maharashtra, India. Glob Health Sci Pract 2022;10. 10.9745/GHSP-D-22-00050 PMC9622279 36316150

[26] Averbach S, Thomas EE, Kully G, Nazarbegian M, Ghule M, Rabin BA, Understanding feasibility and acceptability of implementation of linking delivery of family planning and infant vaccination care in rural Maharashtra, India: a qualitative study. BMC Pregnancy Childbirth 2023;23:519. 10.1186/s12884-023-05830-z 37454051 PMC10349507

[27] Dixit A, Johns NE, Ghule M, Battala M, Begum S, Yore J, Male-female concordance in reported involvement of women in contraceptive decision-making and its association with modern contraceptive use among couples in rural Maharashtra, India. Reprod Health 2021;18:139. 10.1186/s12978-021-01187-8 34193214 PMC8244175

