# Supplementary material for: Psychometric evaluation of the desire to avoid pregnancy scale in India
Source: Contraception. Author manuscript; Available in PMC 2026 Jun 11. (PMC13254518; doi:10.1016/j.contraception.2025.110940)
Supplement: 1 [file NIHMS2176675-supplement-1.docx]

**Appendix A. Mean response, factor loading, item-total correlation, and α if removed for items in the DAP scale in Maharashtra, India 2020-2022 (N=887)**

|  | **Item** | **Mean (SD)** | **Factor loading** | **Item-test correlation** | **α if item removed** |
| --- | --- | --- | --- | --- | --- |
| 1 | I wouldn’t mind it if I became pregnant in the next 3 months. | 2.87 (1.45) | 0.83 | 0.82 | 0.88 |
| 2 | It would be a good thing for me if I became pregnant in the next 3 months. | 2.88 (1.39) | 0.84 | 0.84 | 0.88 |
| 3 | Thinking about becoming pregnant in the next 3 months makes me feel unhappy. * | 2.13 (1.42) | 0.80 | 0.77 | 0.89 |
| 4 | Thinking about becoming pregnant in the next 3 months makes me feel excited. | 2.89 (1.25) | 0.74 | 0.78 | 0.89 |
| 5 | Becoming pregnant in the next 3 months would bring me closer to my main partner. | 3.07 (1.00) | 0.45 | 0.54 | 0.90 |
| 6 | I want to have a baby within the next year. | 2.78 (1.43) | 0.81 | 0.81 | 0.89 |
| 7 | If I had a baby in the next year, it would be bad for my life. * | 1.37 (1.27) | 0.62 | 0.59 | 0.90 |
| 8 | It would be a positive addition to my life to have a baby in the next year. | 2.59 (1.35) | 0.78 | 0.80 | 0.89 |
| 9 | It would be the end of the world for me to have a baby in the next year. * | 0.88 (0.98) | 0.41 | 0.40 | 0.90 |
| 10 | Thinking about having a baby within the next year makes me smile. | 2.96 (1.02) | -0.40 | -0.34 | 0.92 |
| 11 | Thinking about having a baby within the next year makes me feel stressed out. * | 1.85 (1.43) | 0.84 | 0.79 | 0.89 |
| 12 | I would feel a loss of freedom if I had a baby in the next year. * | 1.26 (1.26) | 0.71 | 0.63 | 0.89 |
| 13 | If I had a baby in the next year, it would be hard for me to manage raising the child. * | 1.81 (1.50) | 0.84 | 0.79 | 0.89 |
| 14 | I would worry that having a baby in the next year would make it harder for me to achieve other things in my life. * | 1.42 (1.35) | 0.77 | 0.69 | 0.89 |

* Items marked with an asterisk were reverse coded.

**Appendix B. Mean response, factor loading, item-total correlation, and α if removed for items in the DAP-13 scale in Maharashtra, India 2020-2022 (n=887)**

|  | **Item** | **Mean (SD)** | **Factor loading** | **Item-test correlation** | **α if item removed** |
| --- | --- | --- | --- | --- | --- |
| 1 | I wouldn’t mind it if I became pregnant in the next 3 months. | 2.87 (1.45) | 0.84 | 0.81 | 0.91 |
| 2 | It would be a good thing for me if I became pregnant in the next 3 months. | 2.88 (1.39) | 0.85 | 0.82 | 0.91 |
| 3 | Thinking about becoming pregnant in the next 3 months makes me feel unhappy. * | 2.13 (1.42) | 0.74 | 0.77 | 0.92 |
| 4 | Thinking about becoming pregnant in the next 3 months makes me feel excited. | 2.89 (1.25) | 0.78 | 0.76 | 0.92 |
| 5 | Becoming pregnant in the next 3 months would bring me closer to my main partner. | 3.07 (1.00) | 0.51 | 0.51 | 0.92 |
| 6 | I want to have a baby within the next year. | 2.78 (1.43) | 0.80 | 0.79 | 0.91 |
| 7 | If I had a baby in the next year, it would be bad for my life. * | 1.37 (1.27) | 0.56 | 0.62 | 0.92 |
| 8 | It would be a positive addition to my life to have a baby in the next year. | 2.59 (1.35) | 0.79 | 0.78 | 0.91 |
| 9 | It would be the end of the world for me to have a baby in the next year. * | 0.88 (0.98) | 0.38 | 0.45 | 0.93 |
| 10 | Thinking about having a baby within the next year makes me feel stressed out. * | 1.85 (1.43) | 0.77 | 0.80 | 0.91 |
| 11 | I would feel a loss of freedom if I had a baby in the next year. * | 1.26 (1.26) | 0.61 | 0.66 | 0.92 |
| 12 | If I had a baby in the next year, it would be hard for me to manage raising the child. * | 1.81 (1.50) | 0.77 | 0.80 | 0.91 |
| 13 | I would worry that having a baby in the next year would make it harder for me to achieve other things in my life. * | 1.42 (1.35) | 0.66 | 0.71 | 0.92 |

* Items marked with an asterisk were reverse coded.

**Appendix C. Distribution of average DAP-13 scale response in Maharashtra, India 2020-2022 (N=887)**

**Appendix D. Separation reliability and Item location for items in the DAP-13 scale in Maharashtra, India 2020-2022 (N=887)**

|  | **Separation Reliability** | **0.93** |
| --- | --- | --- |
|  | **Item** | **Item fit** |
| 1 | I wouldn’t mind it if I became pregnant in the next 3 months. | 0.77 |
| 2 | It would be a good thing for me if I became pregnant in the next 3 months. | 0.67 |
| 3 | Thinking about becoming pregnant in the next 3 months makes me feel unhappy. * | 0.97 |
| 4 | Thinking about becoming pregnant in the next 3 months makes me feel excited. | 0.77 |
| 5 | Becoming pregnant in the next 3 months would bring me closer to my main partner. | 1.50 |
| 6 | I want to have a baby within the next year. | 0.84 |
| 7 | If I had a baby in the next year, it would be bad for my life.* | 1.18 |
| 8 | It would be a positive addition to my life to have a baby in the next year. | 0.89 |
| 9 | It would be the end of the world for me to have a baby in the next year.* | 1.43 |
| 10 | Thinking about having a baby within the next year makes me feel stressed out. * | 0.78 |
| 11 | I would feel a loss of freedom if I had a baby in the next year. * | 0.96 |
| 12 | If I had a baby in the next year, it would be hard for me to manage raising the child. * | 0.78 |
| 13 | I would worry that having a baby in the next year would make it harder for me to achieve other things in my life. * | 0.90 |

* Items marked with an asterisk were reverse coded.

**Appendix E:** **Distribution of responses to DAP scale items in Maharashtra, India 2020-2022 (N=887)**

| Item # | **Item description** | Strongly Agree | | Agree | | Neither agree nor disagree | | Disagree | | Strongly Disagree | |
| --- | --- | --- | --- | --- | --- | --- | --- | --- | --- | --- | --- |
|  |  | % | n | % | n | % | n | % | n | % | n |
| 1 | I wouldn’t mind it if I became pregnant in the next 3 months. | 11.6 | 103 | 14 | 124 | 0.6 | 5 | 23.4 | 208 | 50.4 | 447 |
| 2 | It would be a good thing for me if I became pregnant in the next 3 months. | 9.9 | 88 | 14.2 | 126 | 0.5 | 4 | 28.6 | 254 | 46.8 | 415 |
| 4 | Thinking about becoming pregnant in the next 3 months makes me feel excited. | 7 | 62 | 13.4 | 119 | 1.5 | 13 | 39.8 | 353 | 38.3 | 340 |
| 5 | Becoming pregnant in the next 3 months would bring me closer to my main partner. | 1.7 | 15 | 10.7 | 95 | 3.8 | 34 | 46 | 408 | 37.8 | 335 |
| 6 | I want to have a baby within the next year. | 12.4 | 110 | 13.4 | 119 | 0.8 | 7 | 30.1 | 267 | 43.3 | 384 |
| 8 | It would be a positive addition to my life to have a baby in the next year. | 8.8 | 78 | 21.9 | 194 | 1.8 | 16 | 36.5 | 324 | 31 | 275 |
| 10 | Thinking about having a baby within the next year makes me smile. | 0.6 | 5 | 15.8 | 140 | 2.8 | 25 | 48.5 | 430 | 32.4 | 287 |
|  |  | Strongly Disagree | | Disagree | | Neither agree nor disagree | | Agree | | Strongly Agree | |
|  | **Reverse coded items** | % | n | % | n | % | n | % | n | % | n |
| 3 | Thinking about becoming pregnant in the next 3 months makes me feel unhappy. | 17.1 | 152 | 24.2 | 215 | 4.8 | 43 | 35.3 | 313 | 18.5 | 164 |
| 7 | If I had a baby in the next year, it would be bad for my life. | 29.5 | 262 | 37.2 | 330 | 5.6 | 50 | 21.8 | 193 | 5.9 | 52 |
| 9 | It would be the end of the world for me to have a baby in the next year. | 41.1 | 365 | 42.4 | 376 | 4.5 | 40 | 11.2 | 99 | 0.8 | 7 |
| 11 | Thinking about having a baby within the next year makes me feel stressed out. | 21.3 | 189 | 31.7 | 281 | 1.8 | 16 | 30.8 | 273 | 14.4 | 128 |
| 12 | I would feel a loss of freedom if I had a baby in the next year. | 31.3 | 278 | 42.4 | 376 | 1.9 | 17 | 17.6 | 156 | 6.8 | 60 |
| 13 | If I had a baby in the next year, it would be hard for me to manage raising the child. | 25.8 | 229 | 29 | 257 | 1.1 | 10 | 26.6 | 236 | 17.5 | 155 |
| 14 | I would worry that having a baby in the next year would make it harder for me to achieve other things in my life. | 30.3 | 269 | 37.2 | 330 | 1.7 | 15 | 21.8 | 193 | 9 | 80 |
